# Magnetic Stimulation Drives Macrophage Polarization in Cell to–Cell Communication with IL-1β Primed Tendon Cells

**DOI:** 10.3390/ijms21155441

**Published:** 2020-07-30

**Authors:** Adriana Vinhas, Ana F. Almeida, Ana I. Gonçalves, Márcia T. Rodrigues, Manuela E. Gomes

**Affiliations:** 1 3B’s Research Group, I3Bs—Research Institute on Biomaterials, Biodegradables and Biomimetics, University of Minho, Headquarters of the European Institute of Excellence on Tissue Engineering and Regenerative Medicine, AvePark, Parque de Ciência e Tecnologia, Zona Industrial da Gandra, 4805-017 Barco, Guimarães, Portugal; adrianavinhas@i3bs.uminho.pt (A.V.); ana.almeida@i3bs.uminho.pt (A.F.A.); ana.goncalves@i3bs.uminho.pt (A.I.G.); mrodrigues@i3bs.uminho.pt (M.T.R.); 2 ICVS/3B’s—PT Government Associate Laboratory, 4710-057 Braga, Guimarães, Portugal

**Keywords:** co-cultures, magnetic stimuli, macrophages, human tendon cells, IL-1β, cell communication, inflammation, tendon, regenerative medicine

## Abstract

Inflammation is part of the natural healing response, but it has been simultaneously associated with tendon disorders, as persistent inflammatory events contribute to physiological changes that compromise tendon functions. The cellular interactions within a niche are extremely important for healing. While human tendon cells (hTDCs) are responsible for the maintenance of tendon matrix and turnover, macrophages regulate healing switching their functional phenotype to environmental stimuli. Thus, insights on the hTDCs and macrophages interactions can provide fundamental contributions on tendon repair mechanisms and on the inflammatory inputs in tendon disorders. We explored the crosstalk between macrophages and hTDCs using co-culture approaches in which hTDCs were previously stimulated with IL-1β. The potential modulatory effect of the pulsed electromagnetic field (PEMF) in macrophage-hTDCs communication was also investigated using the magnetic parameters identified in a previous work. The PEMF influences a macrophage pro-regenerative phenotype and favors the synthesis of anti-inflammatory mediators. These outcomes observed in cell contact co-cultures may be mediated by FAK signaling. The impact of the PEMF overcomes the effect of IL-1β-treated-hTDCs, supporting PEMF immunomodulatory actions on macrophages. This work highlights the relevance of intercellular communication in tendon healing and the beneficial role of the PEMF in guiding inflammatory responses toward regenerative strategies.

## 1. Introduction

Tendon pathologies are among the most debilitating orthopedic problems due to poor tissue response to currently available treatments, affecting both elderly and active populations. Tendon lesions are often multifactorial and frequently occur due to trauma, overuse activities, and aging. However, unresolved inflammation seems to be a transversal stage in all tendon disorders. Persistent inflammatory events may lead to chronic/degenerative changes in tendon structure, impairing tissue functionality and increasing the risk of re-injury [1,2]. It is thus imperative to create new strategies to improve tendon repair and address therapeutic benefit for patients with tendon diseases [3].

In damaged tendons, tenocytes are exposed to leukocytes and inflammatory mediators. Macrophages (Mφ), in particular, undergo distinct phenotypes and specific functional changes in response to local microenvironment signals to regulate tissue repair and regeneration [4]. In an initial response, activated macrophages (M1 subtype) are responsible for the release of pro-inflammatory cytokines (e.g., TNFα, IL-1β, IL-6, IL-12, IL-23), trophic factors as chemokines and signaling molecules into the repair site in order to promote inflammation, extracellular matrix (ECM) degradation, and debris clearance. In the later stages, pro-regenerative macrophages (M2 subtype) coordinate ECM deposition, secreting anti-inflammatory mediators (e.g., IL-4, IL-10, IL-13, TGFβ) and cell-attracting chemokines (e.g., CCL17, CCL22, and CCL24) to attenuate and assist the resolution of inflammatory events and promote tendon repair [5,6,7].

Although immune cells are necessary for tendon repair, their persistent activation can result in incomplete resolution of inflammation [8] and may lead to chronic injury [9]. Recent studies indicated that the adaptive and innate immune systems work with tissue resident cells to coordinate tissue repair [10], suggesting that the mechanisms to counteract inflammatory stimuli may be insufficient in natural tendon injury. Consequently, this limited intrinsic response creates new opportunities to investigate the interplay of immune cells and human tendon cells (hTDCs) envisioning new molecular and cellular treatment possibilities. Thus, in this work, we approach co-culture methods of Mφ and hTDCs to investigate intercellular communication and the interactions mediated by secreted messengers in fine-tuning cell responses after hTDCs exposure to pro-inflammatory cues (IL-1β supplemented medium), likely present in tendon injuries.

Reinforcing the physiological role of intercellular communication in the tendon, recent works demonstrated that mesenchymal stem cells (MSCs) could “educate” macrophages via paracrine mechanisms into M2 phenotype to improve tendon healing [7] and that tenocytes isolated from ruptured tendons responded to a soluble pool of inflammatory factors, being able to influence macrophage polarization [11].

Together with complex dynamics of regulatory signals, modulation of tendon immune biology events, including the switch of macrophage functional phenotypes, may be assisted by external triggers [12]. Research has shown that the pulsed electromagnetic field (PEMF), a biophysical form of stimulation, accelerates cell differentiation, increases deposition of collagen, and modulates the activation of cell surface receptors, thereby holding relevant contributions for the re-establishment of homeostatic cell functions [13]. Thus, magnetic-based approaches have the potential to become an alternative treatment modality for regenerative therapies [14].

Low-frequency PEMF can modulate inflammatory response in MSCs and macrophages [13,15], and specific parameters of PEMF were also shown by our group to modulate the cytokine profile of IL-1β conditioned tendon cells [16]. Therefore, magnetic stimulation may play an important role in the inflammatory process of injured tissues, resulting in enhanced functional recovery and support for tissue regeneration. Hence, in this work, we also studied the influence of the PEMF to modulate the interactions occurring between macrophages and IL-1β treated tendon cells.

## 2. Results

### 2.1. The hTDCs and Macrophages Phenotype Profile in Response to Co-Cultures Systems and to PEMF Stimulus

The cell-to-cell contact model was chosen for this study, as it enables both secretory and direct contact forms of communication, more closely preserving the intercellular networking and physiological behavior of populations. Since we were also exploring the effect of magnetic stimuli and IL-1β conditioned cells in the modulation of the heterotopic cell crosstalk, we investigated indirect co-cultures to confirm if cell metabolic activity and the gene transcripts of tendon and immune-associated markers were not more affected by paracrine effects rather than cellular interactions. The co-culture models were subjected to either the control basal medium (Ctrl) or medium supplemented with IL-1β cytokine (IL-1β). Both Ctrl and IL-1β treated cultures show similar metabolic activity profiles (Figure S1, supplementary file), independently of cell–cell contact degree or PEMF stimulus. However, the indirect co-cultures show significantly lower values in comparison to direct co-cultures (*p* > 0.05 only for the Ctrl condition).

#### 2.1.1. Impact of Co-Cultures in the Transcript Profiles of Tendon- and Immune-Related Genes

**Figure 1:**
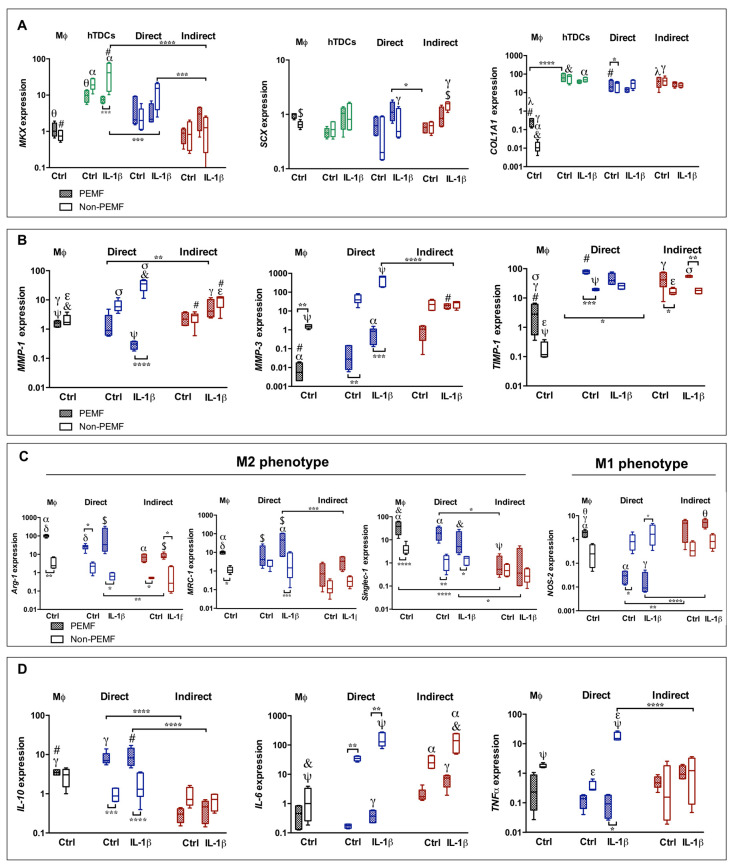
Pulsed electromagnetic field (PEMF) actuation affecting tenogenic and macrophage phenotype in co-culture models 24 h after magnetic (PEMF) actuation. Analysis of the gene expression of (**A**) tendon-related genes (*MKX*, *SCX*, and *COL1A1*), (**B**) extracellular matrix (ECM) remodeling genes (*MMP-1*, *MMP-3*, and *TIMP-1*), (**C**) markers associated to macrophage phenotype (*Arg-1*, *MRC-1*, *Singlec-1*, and *NOS-2*), and (**D**) cytokines (*IL-10*, *IL-6*, and *TNFα*). The expression of target genes was normalized against *GAPDH*. Data analysis was performed using two-way ANOVA followed by multiple comparisons tests (GraphPad Prism), (*n* = 3, three experimental replicates from three biological replicates). Control condition (Ctrl) refers to the absence of IL-1β treatment. Statistically significant differences are shown with different degrees of confidence. Symbols *, γ, $, denote statistical differences for *p* < 0.05; **, α, θ, σ for *p* < 0.01; ***, #, δ for *p* < 0.001; and ****, ψ, &, ε for *p* < 0.0001, respectively.

A tendency for upregulation of tendon-related markers, namely, Mohawk (*MKX)*, Scleraxis *(SCX)*, and Collagen type 1 (*COL1A1)* was observed in both co-culture systems (Figure 1A). Unlike *MKX*, *SCX*, and *COL1A1* expression in co-cultures are similar to the ones in the hTDCs group (*p* > 0.05). Although *MKX* expression in co-cultures is lower than those in the hTDCs group, *MKX* levels are higher in direct co-cultures when compared to indirect co-cultures (direct IL-1β non-PEMF, *p* < 0.001; indirect IL-1β non-PEMF, *p* < 0.0001). As expected, the expression of tenogenic genes in macrophage group is basal or downregulated.

The expression of *MMP-1* and *MMP-3* shows distinctive behavior in direct and indirect co-cultures. In the former, the upregulation of MMPs seems to be associated to PEMF/non-PEMF conditions, while in indirect co-cultures, the differences among the conditions studied are not so evident (*p* > 0.05). In particular, *MMP-1* shows increased expression in direct co-cultures conditioned to IL-1β (non-PEMF, *p* < 0.001) (Figure 1B). Interestingly, the *TIMP-1* expression is upregulated in both types of co-culture, and it is not significantly affected by IL-1β (Figure 1B).

The macrophage genetic profile was considered by measuring the relative expression of phenotype-switch genes *Arg-1*, *MRC-1*, *Singlec-1*, and *NOS-2* (Figure 1C). In both types of co-culture, M2-like markers *Arg-1*, *MRC-1*, and *Singlec-1* show a similar pattern to the ones of macrophages group, despite the incremental expression in PEMF conditions for all these genes. The *NOS-2* expression, a M1-like marker, shows increased values in indirect co-cultures, independently of the culture medium or PEMF stimulation.

The profile of cytokine genes was also studied (Figure 1D). The anti-inflammatory *IL-10* showed lower levels in indirect cultures. Nevertheless, the pro-inflammatory *IL-6* was upregulated in all conditions of indirect cultures, but not for PEMF conditions in direct co-cultures. With the exception for direct cultures treated with IL-1β, the *TNFα* expression tends to be downregulated.

Overall, the genetic expression in direct systems show values closer to hTDCs or macrophage control groups, and with distinctive patterns among the conditions studied. These outcomes suggest that the physical communication between cells enable contact-mediated signals that are not perceived via soluble factor–cell signaling.

#### 2.1.2. Effect of the PEMF on Gene Transcription Levels in Co-Culture Systems

The effect of the PEMF was also approached in direct and indirect communication between hTDCs and macrophages.

In terms of tenogenic gene expression, *MKX*, *SCX*, and *COL1A1* were upregulated, independently of the PEMF (Figure 1A). An exception is *MKX* in the IL-1β treated hTDCs group (*MKX* shows lower values under PEMF application, *p* < 0.01 and *COL1A1* is enhanced in Ctrl conditions in direct co-cultures, *p* < 0.0001).

In direct cultures, a dissimilar pattern of the *MMPs* and *TIMP-1* in PEMF and non-PEMF conditions is observed. PEMF actuation decreases the expression of *MMP-1* and *MMP-3* (Figure 1B) and upregulates *TIMP-1*. In the case of *MMP-1*, this effect is especially noted in direct cultures treated with IL-1β (*p* < 0.0001). Interestingly, PEMF causes a downregulation of *MMP-3* in direct cultures and in macrophage group, while in non-PEMF conditions, *MMP-3* is upregulated, independently of IL-1β treatment (Ctrl group, *p* < 0.01 and IL-1β, *p* < 0.001).

Relatively to *TIMP-1*, the gene was upregulated in all conditions, with the highest expression found in direct co-cultures actuated by PEMF and without IL-1β treatment (indirect cultures, *p* < 0.05 and macrophage group under PEMF, *p* < 0.001). These results comply with the upregulation of *COL1A1*, suggesting an anabolic effect of the PEMF over the synthesis of collagen type I.

*Arg-1*, *MRC-1*, and *Singlec-1* (M2) were upregulated in direct cultures with higher expression in the PEMF condition in both Ctrl and IL-1β treated cells (*p* < 0.05) (Figure 1C). In indirect co-cultures, the PEMF effect only affects *Arg-1* expression (*p* < 0.05). However, expression of *NOS-2* (M1) was downregulated in direct co-cultures under the PEMF in comparison to indirect cultures (Ctrl PEMF, *p* < 0.01; IL-1β PEMF, *p* < 0.0001; and macrophage group, *p* < 0.01).

These outcomes suggest that genes associated to macrophage polarization can be modulated by an external magnetic stimulus. Moreover, there is a combinatorial effect between the PEMF and the interplay of macrophages and tendon cells in the regulation of M1 and M2 genes in comparison to macrophage control group (Ctrl PEMF, *p* < 0.01; IL-1β PEMF, *p* < 0.01; *NOS-2* and IL-1β PEMF, *p* < 0.05; and *MRC-1, p* < 0.01).

Correlating with the results obtained for macrophage phenotype in Figure 1C, the transcript levels of *IL-10* were tenfold higher in PEMF-actuated cultures (in comparison with indirect co-cultures, *p* < 0.0001 and with macrophage group, *p* < 0.05). Interestingly, *IL-6* and *TNFα* expression were downregulated in PEMF-exposed co-cultures (direct) and in the macrophage group, while non-PEMF cultures induced an exponential increment in IL-6 expression, especially in IL-1β treated cells (Ctrl and IL-1β non-PEMF, *p* < 0.01).

With few exceptions (enhanced *MMP-1* and *TNFα* expression in non-PEMF direct cultures), we could not find significant differences in macrophage and hTDCs cultured in Ctrl or IL-1β supplemented media. Despite the fact that IL-1β induces the synthesis of inflammatory cues [17], the interaction between hTDCs and immune cells leads to anti-inflammatory signals that decrease the impact of IL-1β in the transcript of inflammatory-associated genes.

In sum, PEMF seems to play a modulatory action on macrophage-, cytokine-, and ECM remodeling-associated genes, especially in cell-to-cell contact cultures, without a significant interference in the genetic expression of tenogenic markers.

### 2.2. Synthesis of Proteins and Soluble Factors in hTDCs and Macrophages Co-Cultures

Cell-to-cell contact provides a more natural environment between populations. Additionally, under PEMF actuation, direct contact communication seems to be a more effective approach in the education of tendon cells and macrophages at the genetic level. At the protein level, the percentage of cocultured cells positive for SCX and MKX were similar in all conditions studied corroborating the genetic analysis, although the number of cells producing collagen (COL1, COL3) tended to be lower in control conditions (non-PEMF and without IL-1β) (Figure S2(Ai), supplementary file). Furthermore, when cells are treated with IL-1β and magnetically stimulated, there is a tendency to increase the number of cells expressing CD169 (M2) in comparison to other conditions and to macrophage control group (*p* > 0.05), and to decrease the number of cells positive for CD163 (M2). Interestingly, the number of cells expressing CD80 (M1) also tended to decrease in co-cultures in comparison to macrophage control group (Figure S2(Aii), supplementary file).

**Figure 2:**
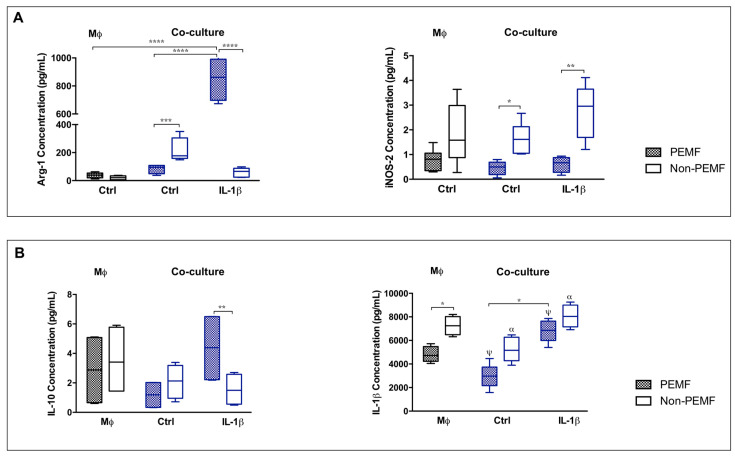
IL-1β and magnetic stimulation differently affect cell contact co-cultures. (**A**) Arg-1 and iNOS, and (**B**) IL-10 and IL-1β quantification in culture medium 24 h after PEMF and IL-1β stimulation. Data analysis was performed using two-way ANOVA followed by multiple comparisons tests (GraphPad Prism), (*n* = 3, three experimental replicates from three biological replicates). Statistically significant differences are shown with different degrees of confidence. Symbols: * for *p* < 0.05; **, α for *p* < 0.01; *** for *p* < 0.001; and ****, ψ for *p* < 0.0001, respectively.

Then, we focused our studies in the production and secretion of Arg-1 and iNOS. Arg-1 (Arginase 1) and iNOS (nitric oxide synthase) are two key enzymes of the arginine metabolism, related to the macrophage polarization into M2 and M1, respectively (Figure 2A).

The concentration of Arg-1 was higher in IL-1β PEMF condition (*p* < 0.0001) in comparison to Ctrl PEMF and to macrophage group. Contrariwise, secreted iNOS was significantly decreased on PEMF-stimulated cells, with or without IL-1β treatment (Ctrl, *p* < 0.05; IL-1β, *p* < 0.01). Both secreted forms of Arg-1 and iNOS follow the pattern of *Arg-1* and *NOS-2* expression (Figure 1C). These results demonstrate that PEMF modulates the gene expression and the release of macrophage associated enzymes supporting a M2-like phenotype on hTDCs–macrophage cultures.

Hence, we investigated the impact of PEMF in cytokine production (Figure 2B). IL-10 release was significantly increased under PEMF in IL-1β treated co-cultures when compared to non-PEMF condition (*p* < 0.01). Meanwhile, soluble IL-1β was diminished in PEMF-actuated cells (in both types of co-culture and macrophage group, *p* < 0.05), proposing that PEMF modulates the production of these cytokines.

**Figure 3:**
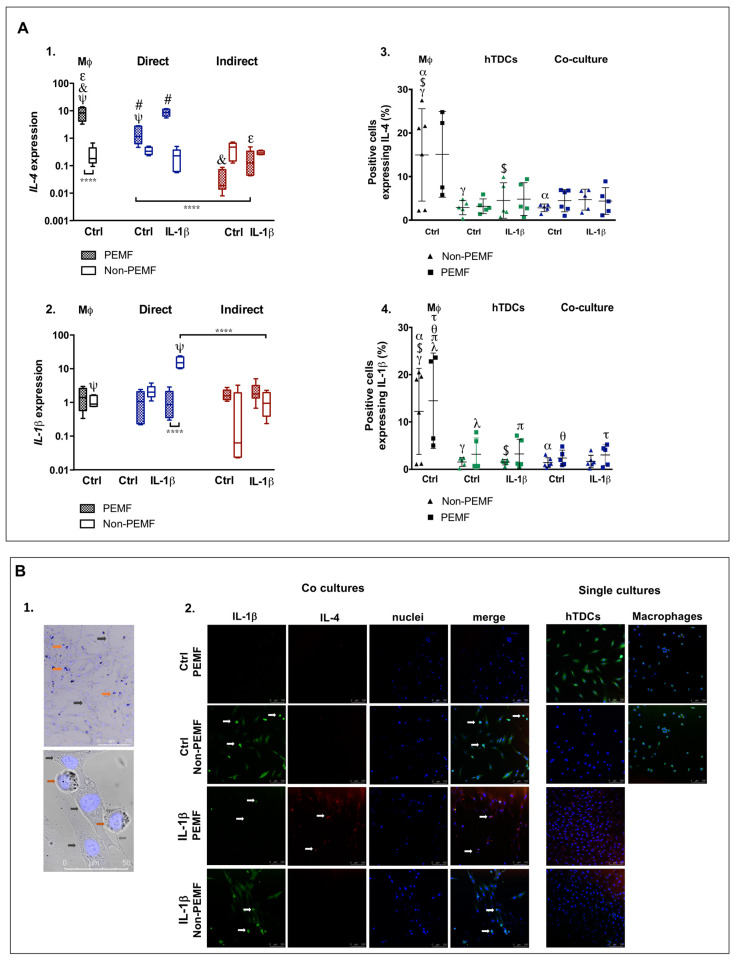
IL-1β influences pro- and anti-inflammatory markers in co-cultures under magnetic stimulation (PEMF.) (**A1**,**A2**) Genetic expression of *IL-4* and *IL-1β* upon PEMF and IL-1β stimulation by RT-PCR analysis. (**A3**,**A4**) Flow cytometry analysis of intracellular cytokines, IL-4, and IL-1β in co-cultures and in hTDCs and Mφ (controls) upon PEMF and IL-1β stimulation. The white arrows identify THP-1 cells in co-cultures by their round-shaped and single-cell morphology. (**B1**) Microscopic images of the dissimilar morphology of macrophages and hTDCs, enabling their identification in co-culture systems. THP-1 derived macrophages show a round and single-cell morphology (red arrows), while hTDCs are larger elongated fusiform cells (black arrows). The physical contact between THP-1 and hTDCs is evidenced by the gray arrow (bottom image). Phase contrast images counterstained with DAPI (×10; scale bar 250μm, and x63; scale bar 50 μm, respectively). (**B2**) Immunofluorescence images of IL-1β (green), IL-4 (red), nuclei (DAPI, blue), and merged image 24 h after PEMF stimulation on co-cultures (confocal microscopy ×20, scale bar 100 μm). Control condition (Ctrl) refers to the absence of IL-1β. Statistically significant differences are shown with different degrees of confidence. Symbols γ, $, λ, τ, π denote statistical differences for *p* < 0.05; α, θ for *p* < 0.01; # for *p* < 0.001; and ****, ψ, &, ε for *p* < 0.0001.

When investigating IL-4 and Il-1β produced by co-cultured cells, genetic expression demonstrates upregulation of *IL-4* but not of *IL-1β* when co-cultures were treated with IL-1β and stimulated with PEMF (in comparison to the Ctrl conditions, *p* < 0.001) (Figure 3A1,A2). In addition, the percentage of cells expressing intracellular IL-4 and IL-1β was similar in all conditions studied, indicating that IL-1β treatment or PEMF stimulus do not interfere with the production of these cytokines (Figure 3A3,A4).

To corroborate these results and assess the contributions of hTDCs and macrophages to the expression of these cytokines, immunofluorescence for IL-1β and IL-4 was assessed (Figure 3B). hTDCs and macrophages can be identified in co-cultures through distinctive morphologic characteristics (Figure 3B1). More specifically, THP-1 derived macrophages have a single-cell morphology, round-shaped with vesicle-like structures in the cytoplasm and are considerably smaller than hTDCs. On the other hand, hTDCs are fusiform elongated cells that form networks with nearby hTDCs. 

Cells co-cultured under PEMF-stimulated conditions in combination with IL-1β primed hTDCs show stronger signal intensity in IL-4 (Figure 3B2). In single cell control groups, both hTDCs and macrophage contribute for IL-1β, but the signal of Il-4 is only visible in Il-1β treated hTDCs, with or without PEMF stimulation. These outcomes suggest that hTDCs respond differently to IL-1β in the presence of macrophages, highlighting the role of communication networks in the regulation of inflammatory cues.

### 2.3. Intercellular Communication between Tendon Cells and Macrophages on Pro-Inflammatory Environment

**Figure 4:**
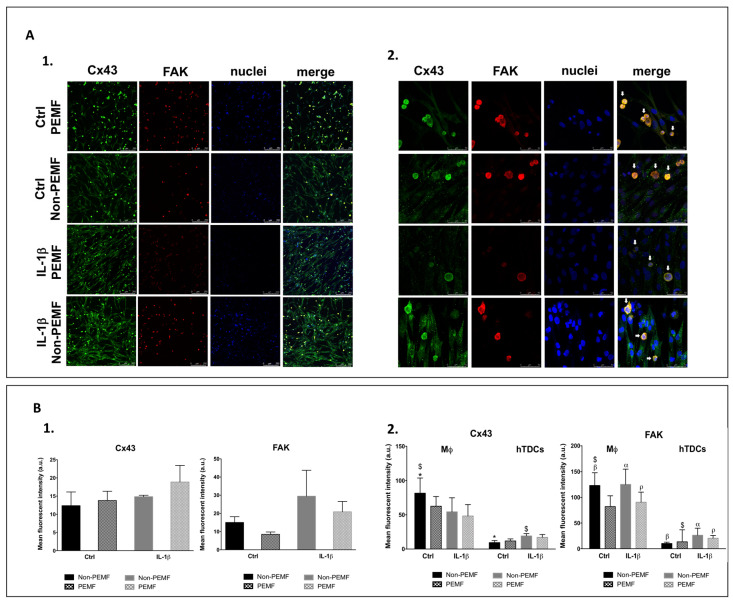
Intercellular communication in co-cultures under the PEMF. (**A1**,**A2**) Confocal microscopy images of Cx43 (green), FAK (red), nuclei (DAPI, blue), and merged image 24 h after PEMF stimulation and IL-1β treatment (confocal microscopy ×10 and ×63, scale bars 250 μm and 50 μm, respectively). The white arrows identify THP-1 cells by their round-shaped and single-cell morphology. (**B1**) Mean fluorescence intensity of Cx43 and FAK on co-cultures. (**B2**) The contribution of macrophage and hTDCs to the mean fluorescence intensity was assessed measuring the signal intensity in macrophages and subtracting this value to the raw image to obtain the hTDCs signal. Control condition (Ctrl) refers to the absence of IL-1β. Data on the graphs are presented as mean ± SEM (*n* = 3, three experimental replicates from three biological replicates) and data analyzed using one- and two-way ANOVA followed by multiple comparisons tests. Statistically significant differences are shown as α, $, *, *p* for *p* < 0.05; β, for *p* < 0.01.

To more comprehensively investigate how macrophages and IL-1β conditioned hTDCs establish cell–cell contacts in the presence of magnetic cues, migration assays (Figure S3, supplementary file) and the involvement of connexin 43 (Cx43) and focal adhesion kinase (FAK) were assessed (Figure 4A1,A2). Cx43 is a protein associated with cell migration and to cell–cell communication in tendon [18], while FAK integrates mechanosensory protein complexes [19] that respond to extracellular stimuli and regulate cellular responses, including cell migration and proliferation [20]. IL-1β treatment on hTDCs seems to stimulate the collective mobility of cells, with masses of both cells spreading into the void area. The migratory cells’ behavior seems not to be affected by PEMF.

Both Cx43 and FAK were expressed in all conditions studied (Figure 4A1). Although Cx43 is detected in both cell types, FAK is predominantly detected in macrophages (Figure 4A1,A2). Overall, Cx43 signal intensity tends to increase with IL-1β treatment and PEMF stimulation (*p* > 0.05), while FAK seems to be more influenced by IL-1β treatment (*p* > 0.05) (Figure 4B1). However, when Cx43 and FAK are analyzed considering each of the cell types present in the co-culture, we observed that the Cx43 signal is significantly increased in macrophages in non-PEMF conditions and without IL-1β treatment in comparison to hTDCs (*p* < 0.05) (Figure 4B2). In addition, the mean fluorescence intensity of FAK in macrophages is significantly increased in all conditions in comparison to hTDCs (*p* < 0.05), except when cells are cultured under PEMF stimulation without IL-1β treatment (*p* > 0.05) (Figure 4B2). Altogether, these results suggest that macrophages are active players in the intercellular communication with hTDCs, which may be mediated by FAK signalling.

## 3. Discussion

Biological functions and homeostasis rely on complex cell responses to microenvironmental stimuli. The articulated interplay of immune cells and resident cell population is critical for timely and spatial regulation to achieve proper healing and stimulate regeneration [10]. Although inflammatory cues are necessary to trigger the repair mechanisms, if perpetuated, pro-inflammatory signals can impair regenerative response and produce a deleterious effect on tendon functionality [21].

The inflammatory response is accompanied by an increasing population of macrophages at the injury site. One of the most powerful pro-inflammatory factors released by these cells is IL-1β that helps to set an inflammatory milieu to which nearby cells respond to [22]. We have previously established an inflammatory model with IL-1β to stimulate tendon cells to exhibit a pro-inflammatory profile associated with tendon injury environments [16]. However, in this work, we aimed to assess whether “inflamed” tendon cells could communicate with immune cells and provoke a macrophage phenotype switch. For that, we studied co-cultures between tendon cells exposed to IL-1β and macrophages. Moreover, we investigated the modulatory effect of PEMF on macrophage and tendon cells interactions, using the parameters identified in our previous work [16].

The upregulation of tenogenic-related markers in both co-cultures suggests that hTDCs maintain their lineage identity, which is not affected by the direct or indirect contact with immune cells or to the exposure to PEMF.

Collagen I is the main component of connective tissues and its degradation by MMPs [23,24] is crucial for tendon repair. As PEMF supports the decrease of MMPs and increase of *TIMP-1* expression, *COL1A1* expression is not compromised, meaning that PEMF can positively actuate on ECM turnover balancing the expression of MMPs and *TIMP-1*, displaying a regulatory action over ECM in tendon healing.

The external stimulation provided by PEMF-affected macrophage polarization toward M2, with the upregulation of *Arg-1*, *MRC-1*, and *Singlec-1*, especially in cell-to-cell contact cultures. This result is in accordance to published studies that have demonstrated the ability of magnetic forces to stimulate Arg-1 in peritoneal Mφ macrophages [25,26]. Conversely, the M1 markers are downregulated in direct cultures stimulated by PEMF.

Another important outcome is that PEMF seems to overpower the signals provided by IL-1β treated hTDCs, highlighting the potential immunomodulatory role of PEMF in tissue repair and regenerative strategies. Moreover, PEMF seems to exert a dual cell action; on macrophages, switching their functional phenotype, and on IL-1β treated cells, antagonizing the pro-inflammatory signals. The impact on both cell types is likely to contribute to improve the healing response and may justify the differential response between direct and indirect cultures.

Not only are the M1/M2 associated genes influenced by the PEMF, but the profile of cytokine genes is influenced as well. Under PEMF, *IL-10* was upregulated, while *IL-6* and *TNFα* were downregulated in direct cultures exposed to PEMF. The balance toward anti-inflammatory cues also supports the immunomodulatory potential of PEMF, and correlates with outcomes reported in the literature [27].

Taken together, cell–cell signaling combined with the PEMF modulates and guides macrophages’ fates toward the M2 phenotype, contributing to regulation and decreasing the influence of secreted pro-inflammatory cues. Macrophages regulate arginine metabolism via iNOS and Arg-1 with important functional responses for healing [28]. Additionally, Arg-1, inducible by IL-4, leads to the production of ornithine that assists cell proliferation and repair of damaged tissue [29]. Our results point a preferential selection for M2/Arg-1 versus M1/iNOS favoring repair signaling. Overall, PEMF positively affects the gene expression and the release of cytokines on direct cultures, supporting previous results and the immunomodulatory role of PEMF on macrophage functional responses. Due to the relevance of cell-to-cell contact in the exchange of information between macrophages and hTDCs, we investigated a possible involvement of Cx43 and FAK on cell migration and in mediating networks of inflammatory factors. Being a transmembrane protein, Cx43 participates in the flow of data between the intracellular and extracellular compartments [30]. Cx43 is expressed in both tenocytes and immune cells and can be upregulated by inflammatory influencers as TNFα and IFN-y [31]. Moreover, Cx43 is involved in cell migratory phenomena [31,32], including endothelial cells migration during wound repair [33], and affects cell–cell contact facilitated interactions [34]. In our work, IL-1β treatment influenced the cell mobility between macrophages in comparison to hTDCs. Although an increase of Cx43 has been reported to participate in inflammation [31], we did not observe this behavior in our study. The increment in Cx43 signal in macrophages may be associated to specific functions of these cells in immunity or to the interaction with hTDCs. It is likely that the communication between tendon cells and macrophages actuated by PEMF may also privilege other communication channels. FAK was shown to be highly expressed by macrophages and tends to decrease in PEMF conditions. FAK activation in macrophages seems to be independent of IL-1β primed hTDCs, but may be associated to sensing and transmission of magnetic stimulation to immune cells.

Overall, we showed that magnetic stimulation influences the intercellular communication of tendon cells and macrophages, holding immunomodulatory action over macrophages and stimulating M2 phenotype, in which FAK signaling may be involved.

## 4. Materials and Methods

### 4.1. Isolation and Cell Expansion of Human Tendon-Derived Cells

hTDCs were isolated from surplus tissue samples collected from patients undergoing orthopedic reconstructive surgeries under protocols previously established with Hospital da Prelada (Porto, Portugal) and with informed consent of the patients. The content of the written informed consent and related procedures were reviewed and approved by the Hospital Ethics Committee (P.I. N.º 005/2019).

Following a previous established protocol [35,36,37], tendon samples were minced and digested in an enzymatic solution of collagenase I (0.1%, Sigma-Aldrich, C6885, Saint Louis, MO, USA) with 2M CaCl_2_ (1:1000, VWR, Darmstadt, Germany) and 1% bovine serum albumin (BSA) (Sigma-Aldrich, Saint Louis, MO, USA) for 1h at 37 °C under agitation. After incubation, digested samples were filtered and centrifuged three times at 1200 rpm for 5 min, and the supernatant was discarded. Isolated hTDCs were expanded in basic culture medium composed of α-MEM (A-MEM; Invitrogen, Life Technologies Limited, Paisley, UK) supplemented with 10% fetal bovine serum (FBS) (Alfagene, Life Technologies Limited, Paisley, UK) and 1% antibiotic/antimicotic solution (A/A) (Alfagene, Life Technologies Limited, Paisley, UK) in a humidified 5% CO_2_ atmosphere. hTDCs from passage 2 to 4 were used to perform all the assays.

### 4.2. Macrophage Culture and Differentiation

THP-1 cells, a human monocytic cell line that is extensively used to study monocyte/macrophage function and biology, was cultured and expanded in RPMI culture medium (Sigma-Aldrich, Saint Louis, MO, USA), supplemented with 1% A/A in humidified 5% CO_2_ atmosphere. THP-1 derived macrophages were differentiated with 100nM phorbol 12-myristate-13-acetate (PMA, Sigma-Aldrich, Saint Louis, MO, USA) for 24 h, followed by 24 h cultivation with PMA-free medium. Non-attached cells were removed by aspiration and the adherent THP-1 derived macrophages were washed three times with RPMI and further expanded in RPMI medium.

### 4.3. Establishment of Co-Cultures Systems

**Figure 5:**
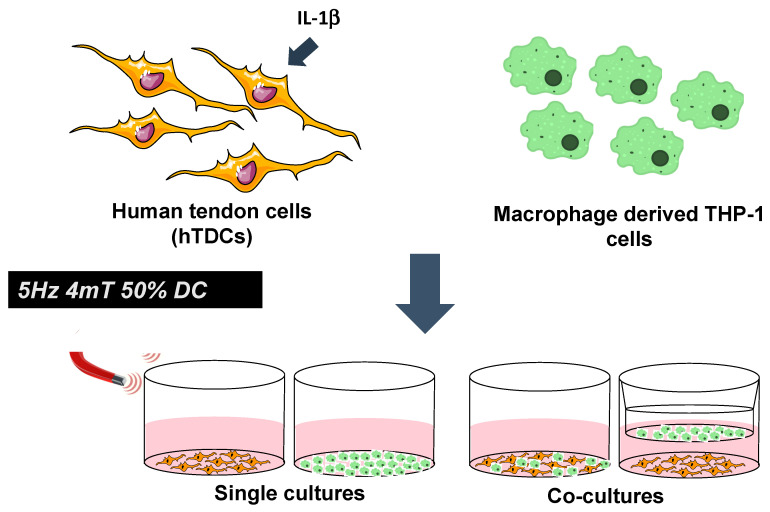
Magnetic modulation of intercellular communication between macrophage and tendon cells treated with IL-1β using co-culture systems. Single cultures were considered as experimental controls. A magnetic stimulation regimen was applied at 5 Hz, 4 mT, and 50% duty cycle.

In vitro co-cultures were established to explore potential crosstalk between macrophages and hTDCs under an inflammatory environment. hTDCs (10,000 cells/cm^2^) were seeded onto 24-well plates (BD Biosciences, San Jose UK) and treated with IL-1β (1 ng/mL, Alfagene, Life Technologies Limited, Paisley, UK) for 24 h to induce inflammatory cues in hTDCs, as previously reported [16]. Co-cultures were established by culturing the macrophages (10,000 cells) either (i) on top of seeded hTDCs (direct cultures) or (ii) in the chamber of a Transwell^TM^ (pore size: 1µm; Corning, VWR, Darmstadt, Germany) placed over cultured hTDCs (indirect paracrine cultures) (Figure 5). Upon seeding for 24 h, co-cultures were exposed to a PEMF stimulation regimen: 5 Hz, 4 mT, and 50% duty cycle for 1 h, following previous studies by our group [16] using a magnetotherapy device (Magnum XL Pro; Globus Corporation, Codogné,Italy). Co-cultures were further cultured for one day upon PEMF exposure, and then assessed for tendon and macrophage phenotypic markers (transmembranar and surface markers), focal adhesions, and for genetic and secreted cytokine profile. Outcomes were compared with single cultures of hTDCs or macrophages as appropriate.

### 4.4. Characterization of Tendon and Macrophage Phenotype in Co-Culture Systems

#### 4.4.1. RNA Isolation and Gene Expression Analysis

Total RNA was extracted using TRI reagent^®^ RNA Isolation Reagent (T9424; Sigma-Aldrich, Saint Louis, MO, USA) following the manufacturer’s instructions. RNA was quantified using a Nanodrop^®^ ND-1000 spectrophotometer (Wilmington, DE, USA) at 260/280 nm. The first-strand complementary DNA was synthesized from 1 μg of RNA of each sample (qScript^TM^ cDNA Synthesis Kit, Quanta Biosciences, Gaithersburg, MD, USA) in a 20 μL reaction using a Mastercycler^®^ ep realplex gradient S machine (Eppendorf, Hamburg, Germany).

The quantification of the transcripts was carried out by quantitative polymerase chain reaction (qPCR) using the PerfeCTA SYBR Green FastMix kit (Quanta Biosciences, Gaithersburg, MD, USA) following the manufacturer’s protocol, in a Real-Time Mastercycler ep realplex thermocycler (Eppendorf, Hamburg, Germany). The primer sequences (Supplementary Table S1) were designed with Primer 3 software and synthesized by MWG Biotech. The 2^−^^ΔΔ*C*t^ method was used to evaluate the relative expression level for each target gene [38].

The transcript expression of target genes (*MKX*, *SCX*, *COL1A1*, *MMP-1*, *MMP-3*, *TIMP-1*, *Arg-1*, *MRC-1*, *Singlec-1*, *NOS-2*, *IL-10*, *IL-6*, *TNFα*, *IL-4*, *IL-10*, *IL-1β*) was analyzed and normalized to the expression of endogenous housekeeping gene *GAPDH* (glyceraldehyde-3-phosphate dehydrogenase) and then to the samples collected at day 0 (*n* = 3).

#### 4.4.2. Quantification of Secreted Cytokines

The supernatants of single and co-cultures were assessed with respect to cytokine concentrations using commercially available enzyme immunoassay kits for Arg-1 (Arginase-1 Human ELISA Kit, BMS2216; Invitrogen, Carlsbad, California, USA), iNOS (Human Inducible nitric oxide synthase ELISA Kit, MBS723617; Mybiosource, San Diego, CA, USA), IL-1β (Human IL-1β Standard ABTS ELISA Development Kit, 900-K95; Peprotech, Rocky Hill, NJ, USA), and IL-10 (Human IL-10 Standard ABTS ELISA Development Kit, 900-K21; Peprotech, Rocky Hill, NJ, USA) following the manufacturer’s instructions.

#### 4.4.3. Assessment of Intracellular Cytokines in Direct Co-Cultures

The cells retrieved from direct co-cultures of hTDCs and macrophages were treated during 4 h with 10 µg/mL Brefeldin A (ab193369, Abcam, Cambridge, UK) to block cytokine secretion before being trypsinized using TrypLE Express (12605-028; Alfagene, Life Technologies Limited, Paisley, UK), centrifuged, and resuspended in fresh PBS. Afterwards, cells in suspension were incubated with fluorochrome-conjugated antibodies: anti-IL-1β (FITC; ab16168, Abcam, Cambridge, UK) and anti-IL-4 (Phycoerythin; ab95717; Abcam, Cambridge, UK) for 20 min at room temperature protected from light. Cells were then rinsed in PBS and centrifuged for 5 min at 800× *g*.

The cells were resuspended in 500 µL of acquisition buffer and data acquired in a FACSAria III sorter equipped with blue and red lasers (BD Biosciences, Erembodegem-Aalst, Belgium). Cells were identified by forward and side scatter. A minimum of 10,000 cells were acquired and analyzed using FACS Diva version 7 software. Unstained cells were considered as negative controls.

The positive population of cells expressing the markers of interest was expressed in percentage values. Data acquired and analyzed is representative of three independent experiments.

#### 4.4.4. Detection of Inflammatory Mediators in Direct Co-Cultures by Immunofluorescence

Cells were fixed with a solution of 10% (*v*/*v*) neutral buffered formalin (Bio Optica, Milano, Italy) overnight, and kept in PBS at 4 °C until usage. Subsequently, cells were incubated with 0.025% Triton-X100 (Sigma-Aldrich, Saint Louis, MO, USA) in PBS and the blocking step was performed using RTU Normal Horse Serum (RTU Vectastain Kit, PK-7200; Vector, Burlingame, CA, USA). Cells were incubated for 1 h at 4 °C with anti-FAK (phosphor Y397), 1:200 (ab39967, Abcam, Cambridge, UK), or connexin 43/GJA1, 1:100 (ab11370; Abcam, Cambridge, UK), both diluted in antibody diluent with background reducing components (Dako, Santa Clara, CA, USA), followed by incubation for 1 h at room temperature with the secondary antibody (donkey anti-rabbit Alexa Fluor 488, 1:200, Alfagene, Life Technologies Limited, Paisley, UK). The direct co-cultured cells were also incubated with fluorochrome-conjugated antibodies: anti-IL-1β (FITC; ab16168, Abcam, Cambridge, UK) and anti-IL-4 (Phycoerythin; ab95717; Abcam, Cambridge, UK) for 30 min at room temperature protected from light.

Samples were washed three times with PBS before stain with 4,6-Diamidino-2-phenyindole, dilactate (DAPI, 5 μg/μl, D9564; Sigma-Aldrich) for 10min, prepared according to the manufacturer’s instructions. The outcomes of protein detection are representative of three independent experiments.

The detection of immunostained proteins, IL-4, IL-1β, FAK, and Cx43, was assessed by confocal laser scanning microscopy (CLSM, TCS SP8, Leica, Wetzlar, Germany) using 63x magnification objective and LAS X software from Leica. Images were bidirectionally scanned at 400 Hz with Argon (488) and UV (405) lasers.

The semi-quantification of mean fluorescence intensity was performed for FAK and Cx43. A minimum of five images per sample were analyzed from independent experiments. The contribution of macrophage and hTDCs to the mean fluorescence intensity was assessed measuring the signal intensity in macrophages using multiple ROI. The hTDCs signal was calculated subtracting the mean signal intensity of macrophages to the initial raw images using ImageJ software version 1.52a, National Institutes of Health, USA.

### 4.5. Statistical Analysis

Results are expressed as mean ± standard error of the mean. In the case of flow cytometry, results are expressed as mean ± standard deviation. The statistical analysis was performed using GraphPad Prism software (version 6.0, San Diego, CA, USA). Data was obtained from three independent experiments (*n* = 3) analyzed in triplicate and evaluated by one-way or two-way ANOVA followed by Bonferroni post-hoc test for multiple comparison tests. A difference was considered significant with a confidence interval of 95%. Different degrees of confidence, *p* < 0.05, *p* < 0.01, *p* < 0.001, and *p* < 0.0001 are represented by symbols *, α, p, γ, $, λ for *p* < 0.05; **, α, β, θ, σ for *p* < 0.01; ***, #, δ for *p* < 0.001; and ****, ψ, &, ε for *p* < 0.0001.

## 5. Conclusions

This work demonstrated that direct communication established between tendon cells and THP-1 derived macrophages plays an important role in the phenotypic expression of immune cells. To modulate macrophage behavior, the impact of magnetic stimulus in cell–cell contact is more important than tendon cells conditioned to IL-1β. PEMF drives the polarization of macrophage toward a pro-regenerative phenotype, assisting the increment of soluble anti-inflammatory factors into the extracellular milieu and favoring an anabolic action over matrix turnover genes. Overall, PEMF actuation evidences an immunomodulatory role on macrophage behavior co-cultured with hTDCs.

This work provides insights on the dynamics of tendon cells and macrophage communication and supports the relevant contribution of the immunomodulatory actions of magnetic actuation for the development of new treatments and in tendon regeneration strategies.

## Supplementary Materials

Supplementary materials can be found at https://www.mdpi.com/1422-0067/21/15/5441/s1.
