# Supplementary material for: Magnetic Stimulation Drives Macrophage Polarization in Cell to–Cell Communication with IL-1β Primed Tendon Cells
Source: Int J Mol Sci. 2020 Jul 30;21(15):5441. doi: 10.3390/ijms21155441 (PMC7432806; doi:10.3390/ijms21155441)
Supplement: Supplementary file 1 [file ijms-21-05441-s001.pdf]

International Journal of
Molecular Sciences

MDPI

Supplementary data

Table S1. Primers used for real time quantitative RT-PCR analysis.

	Primer Sequence	Accession number
**Human Glyceraldehyde 3-phosphate dehydrogenase (*GAPDH*)**	F - TGTACCACCAACTGCTTAGC<br>R - GGCATGGACTGTGGTCATGAG	NM_002046.4
**Mohawk (*MKX*)**	F – TGTTAAGGCCATAGCTGCGT<br>R – TCGCACAGACACCTGGAAAA	NM_173576.5
**Scleraxis (*SCXA*)**	F – CGAGAACACCCAGCCCAAAC<br>R – CTCCGAATCGCAGTCTTTCTGTC	XM_001717912
**Collagen, Type I, alpha 1 (*COL1A1*)**	F – CGAAGACATCCCACCAATCAC<br>R – GTCACAGATCACGTCATCGC	NM_000088.3
**Human matrix metalloproteinase-1 (*MMP-1*)**	F – ACCTGGAAAAATACTACAACCTGAA<br>R – TTCAATCCTGTAGGTCAGATGTGTT	NM_002421.3
**Human matrix metalloproteinase-3 (*MMP-3*)**	F – CACTCACAGACCTGACTCGG<br>R – AGTCAGGGGGAGGTCCATAG	NM_002422.4
**Human metalloproteinase inhibitor-1 (*TIMP-1*)**	F – CATCCGGTTCGTCTACACCC<br>R – GGATAAACAGGGAAACACTGTGC	NM_003254.2
**Human arginase-1 (*ARG-1*)**	F: GGAAAACCAAGTGGGAGCAT<br>R: TGTGGTTGTCAGTGGAGTGT	ENSG00000118520
**Human mannose receptor C-type 1 (*MRC-1*)**	F: TGCTCTACAAGGGATCGGGT<br>R: ACACGCCAAACAAGAACATGA	ENSG00000260314
**Human sialic acid binding Ig like lectin 1 (*SIGLEC-1*)**	F: CAACTTGCTGCGTGTGGAGA<br>R: TGCCTGATTAGATCCTCCTCGG	ENSG00000088827
**Human nitric oxide synthase 2 (*NOS2*)**	F: GGACATCGCGTGGGTGAA<br>R: TTTATCGCTCGGAGCCTGC	ENSG00000007171

**Human interleukin 10 (*IL-10*)**	F – AAGACCCAGACATCAAGGCG<br>R – AATCGATGACAGCGCCGTAG	NM_000572.2
**Human interleukin 6 (*IL-6*)**	F – AGGAGACTTGCCTGGTGAAA<br>R – GCATTTGTGGTTGGGTCAG	NM_000600.4
**Human tumor necrosis factor (*TNFα*)**	F – ATGTTGTAGCAAACCCTCAAGC<br>R – TGATGGCAGAGAGGAGGTTG	NM_000594.3
**Human interleukin 4 (*IL-4*)**	F – GCACCGAGTTGACCGTAACA<br>R – AGGAATTCAAGCCCGCCAG	NM_000589.3
**Human interleukin 1 beta (*IL-1β*)**	F – TGAGCTCGCCAGTGAAATGA<br>R – AGGAGCACTTCATCTGTTTAGGG	NM_000576.2

S1. Assessment of metabolic activity in single and co-culture systems

The metabolic activity of hTDCs and macrophages as single or co-culture systems were evaluated by MTS assay (CellTiter 96® AQueous One Solution, Promega).

Cells were washed with PBS and incubated with a mix of serum-free culture medium without phenol red and MTS solution (5:1 ratio) for 3h at 37 ºC and 5% CO_2 atmosphere, protected from light. After this period, the supernatant was transferred to a 96-well plate and the absorbance read at 490nm (Synergy™HT, BIO-TEK Instruments, Winooski, Vermont) (Figure S1). Samples were read in triplicates and a blank sample (no cells) was assessed as control of the assay.

Figure S1.

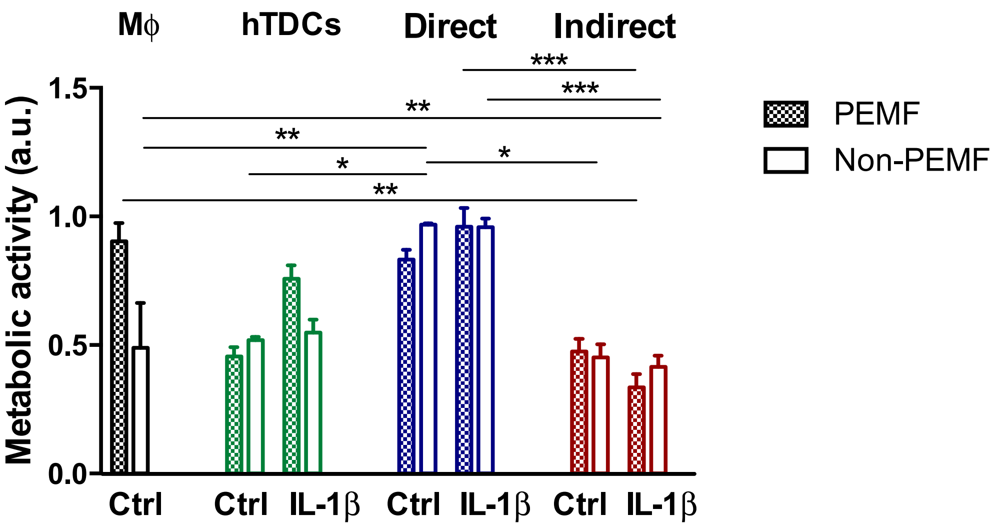

Figure S1. Metabolic activity of hTDCs, macrophages (Mφ) and co-cultures established between hTDCs and macrophage via direct or indirect (transwell) contact 24h after PEMF application (PEMF). IL-1β condition represents hTDCs previously treated with IL-1β. Symbols represent significant differences to control values (Ctrl) that represent the absence of IL-1β treatment. Statistically significant differences are shown as *$p<0.05$; **$p<0.01$; ***$p<0.001$.

S2. Flow cytometry analysis

Cells were trypsinized using TrypLE Express (12605-028; Alfagene, Life Technologies Limited, Paisley, UK), centrifuged and resuspended in PBS. Cells were incubated with antibodies for surface specific markers: CD163 (alexa fluor 647, 8276870, BD Biosciences), CD169 (alexa fluor 647, 565295, BD Biosciences), CD80 (FITC, 9023585, BD Biosciences) and CD68 (E-11) (A1017, santa cruz biotechnology) or with tenogenic markers: Scleraxis (ab58655, Abcam, Cambridge, UK), Mohawk (A83377, Sigma-Aldrich), Collagen I (ab90395, Abcam, Cambridge, UK) and Collagen III (ab7778, Abcam, Cambridge, UK). Cells were incubated for 20 min at RT protected from light before rinsed in PBS and centrifuged for 5 min at 800 g. In the case of unconjugated antibodies, secondary antibodies Alexa Fluor 488 (2072687, Alfagene, Life Technologies Limited, Paisley, UK) and Alexa fluor 594 (2145022, Alfagene, Life Technologies Limited, Paisley, UK) were incubated with cells for 30min at RT protected from light, according to the host species of the primary antibody (Figure S2).

Afterwards, the cells were ressuspended in 500 µL of acquisition buffer and data acquired in a FACSAria III sorter equipped with blue and red lasers (BD Biosciences, Erembodegem-Aalst, Belgium). Cells were identified by forward and side scatter. A minimum of 5,000 cells were acquired and analyzed using FACS Diva version 7 software. Unstained cells were considered as negative controls. The positive cell populations expressing the markers of interest were expressed in percentage values. Data acquired and analysed is representative of three independent experiments.

Figure S2.

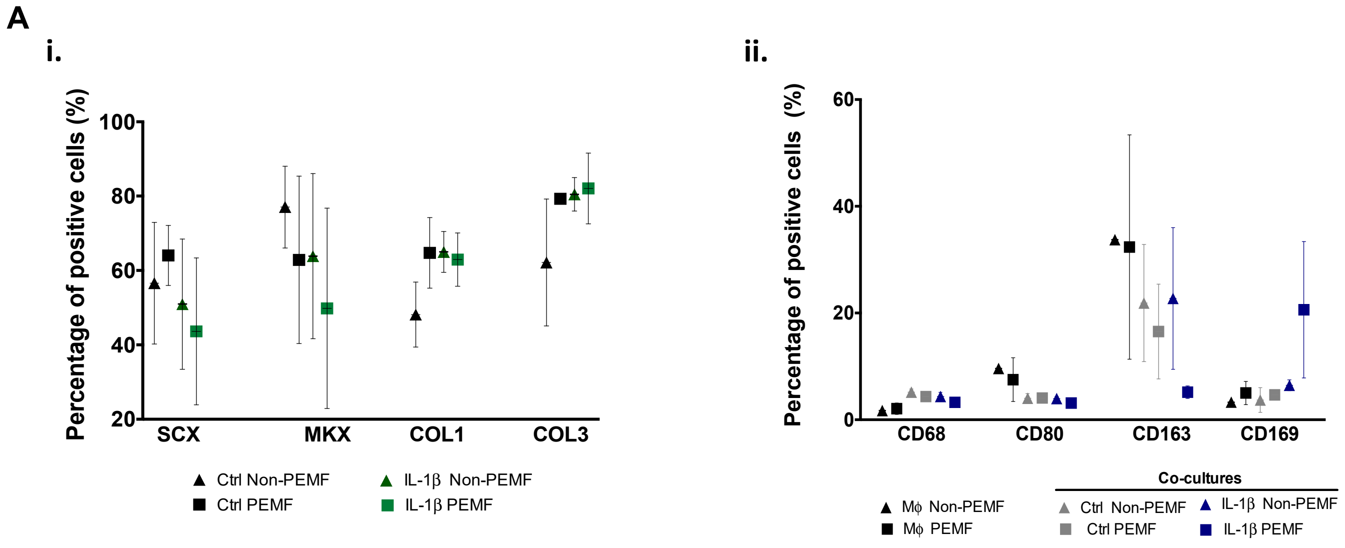

Figure S2. Effect of IL-1β and magnetic stimulation on tenogenic and macrophage markers. Ai) Percentage of cells positive for tenogenic associated markers (SCX, MKX, COL1, COL3).The black color represents control conditions (Ctrl) and green represents IL-1β treatment (IL-1β). Aii) Percentage of cells positive for macrophage phenotypic markers (CD68, CD80, CD163, CD169) while blue and grey represent co-cultures of macrophages and hTDCs with or without IL-1β treatment, repectively. The black color represents macrophage (single cultures). Control condition (Ctrl) refers to the absence of IL-1β.

S3. Cell migration assays

For migration analysis, the hTDCs and macrophages were separately seeded in 2 *well* silicone *insert* with defined cell-free gaps (2 well in μ-dish 35 mm Ibidy, Gräfelfing, German), as previously described [1, 2]. The density of cells inside the well was adjusted to 5,000 hTDCs and macrophages, each. The cell laden inserts were incubated overnight at 37ºC and 5% CO_2, allowing cells to adhere to the bottom of a 24-well well plate (BD Biosciences, UK). After 24h incubation, the debris and non-attached cells were removed rinsing the cells twice with sterile PBS. Cells cultured with RPMI medium were exposed to PEMF stimulation as described before. The mobility of cells was microscopically checked 24h after magnetic stimulation (Figure S3).

The cells were observed in a fluorescent microscope (Carl Zeiss, Germany) and images acquired using a digital camera (AxioCam MRm5) and the Axiovision version 4.8 (Carl Zeiss, Germany) software.

Figure S3.

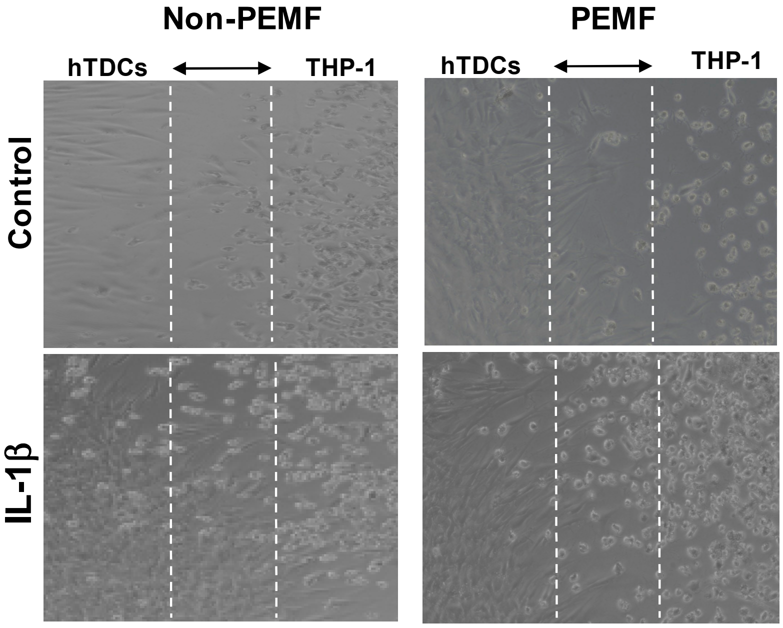

Figure S3. IL-1β induced cell migration in cell contact co-cultures in both non-PEMF and PEMF conditions. Representative images of the cells in 2-well insert assay. Control condition (Ctrl) refers to the absence of IL-1β.

References S3:

1. Yan, T.; Zhang, J.; Tang, D.; Zhang, X.; Jiang, X.; Zhao, L.; Zhang, Q.; Zhang, D.; Huang, Y. Hypoxia regulates mtorc1-mediated keratinocyte motility and migration via the ampk pathway. *PLoS One* **2017**, *12*, e0169155.
2. Caesar, M.; Zach, S.; Carlson, C.B.; Brockmann, K.; Gasser, T.; Gillardon, F. Leucine-rich repeat kinase 2 functionally interacts with microtubules and kinase-dependently modulates cell migration. *Neurobiol Dis* **2013**, *54*, 280-288.
